# Implementation of Multispectral Imaging (MSI) for Microbiological Quality Assessment of Poultry Products

**DOI:** 10.3390/microorganisms8040552

**Published:** 2020-04-11

**Authors:** Evgenia D. Spyrelli, Agapi I. Doulgeraki, Anthoula A. Argyri, Chrysoula C. Tassou, Efstathios Z. Panagou, George-John E. Nychas

**Affiliations:** 1 Laboratory of Microbiology and Biotechnology of Foods, Department of Food Science and Human Nutrition, School of Food and Nutritional Sciences, Agricultural University of Athens, Iera odos 75, 11855 Athens, Greece; eugeniespcheng@gmail.com (E.D.S.); stathispanagou@aua.gr (E.Z.P.); 2 Institute of Technology of Agricultural Products, Hellenic Agricultural Organization “Demeter”, Sof. Venizelou 1, Lycovrissi, 14123 Attica, Greece; adoulgeraki@aua.gr (A.I.D.); anthi.argyri@gmail.com (A.A.A.); soulatassou@gmail.com (C.C.T.)

**Keywords:** poultry products, at-line measurements, multispectral imaging, multivariate data analysis

## Abstract

The aim of this study was to investigate on an industrial scale the potential of multispectral imaging (MSI) in the assessment of the quality of different poultry products. Therefore, samples of chicken breast fillets, thigh fillets, marinated souvlaki and burger were subjected to MSI analysis during production together with microbiological analysis for the enumeration of Total Viable Counts (TVC) and *Pseudomonas* spp. Partial Least Squares Regression (PLS-R) models were developed based on the spectral data acquired to predict the “time from slaughter” parameter for each product type. Results showed that PLS-R models could predict effectively the time from slaughter in all products, while the food matrix and variations within and between batches were identified as significant factors affecting the performance of the models. The chicken thigh model showed the lowest RMSE value (0.160) and an acceptable correlation coefficient (r = 0.859), followed by the chicken burger model where RMSE and r values were 0.285 and 0.778, respectively. Additionally, for the chicken breast fillet model the calculated r and RMSE values were 0.886 and 0.383 respectively, whereas for chicken marinated souvlaki, the respective values were 0.934 and 0.348. Further improvement of the provided models is recommended in order to develop efficient models estimating time from slaughter.

## 1. Introduction

In the last decade, meat consumption has rapidly increased while demand for high-quality meat is expected to continue augmenting as the world population rises. Chicken meat products account for 37% of global meat production due to their low-fat content, affordable price and exclusion of beef and/or pork meat for religious purposes [1]. However, raw poultry products are susceptible to deterioration (short shelf life) and to unpleasant organoleptic attributes during spoilage [2,3]. These facts in tandem with consumers’ demand for fresh meat has led to the development of alternative approaches, such as Process Analytical Technology (PAT), that are considered efficient in predicting quality and freshness in meat products during production [4,5].

PAT is a promising approach for the assessment of products’ quality and it is currently implemented not only in the pharmaceutical industry [6] but also in the food industry [7,8]. The main concept of PAT is the combination of multivariate data derived through real-time (in-, on-, at- line) analytical methods to multivariate data analysis for continuous feedback and information build-up [9]. As analytical techniques of PAT are considered among others spectroscopic methods such as vibrational spectroscopy (FT-IR, NIR, Raman) [10,11,12], hyperspectral and multispectral imaging [13,14,15] and biomimetic sensors (e-nose, e-tongue) [16,17]. Moreover, this innovative approach coupled to microbiological analysis, quality factors and machine learning tools, can permit the understanding of the process, the identification of Critical Control Points (CCPs) and finally the application of a knowledge base to control the process [18,19,20].

According to PAT approach, a potential analysis and sensor have to be able to estimate successfully and rapidly the critical control parameter of interest without the destruction of the product [7]. These requirements are fulfilled in the case of multispectral imaging [21] that combines an optical technique (visible and NIR region) to computer vision in an attempt to obtain spectral and spatial data for the metabolites on the surface of the examined sample. The main advantage over hyperspectral analysis is the fast image acquisition and the usage of simple algorithms for image processing (ROI region) and decision-making [13,22].

In recent years, many researchers have recommended this nondestructive method and several machine learning algorithms for the rapid assessment of meat quality [4,23,24]. Specifically, for poultry products qualitative models were constructed for the classification of intact chicken breast fillets based on three quality grades using hyperspectral analysis [25]. Quantitative and/or qualitative models in the region of visible and near-infrared (400–1700 nm), were able to detect the bacterial population (TVC, *Pseudomonas* spp. and *Enterobacteriaceae*) during spoilage of chicken meat [26,27,28,29]. Other studies involving multispectral imaging were associated with the adulteration of minced beef with chicken meat [30], the presence of fecal contaminants in a poultry line [31,32,33], defects [33,34,35] and tumors on the surface of chicken breasts [36,37].

So far there are limited studies on the implementation of spectroscopic methods during processing at meat industries [38] and the majority is focused on the determination of fat and fatty acids in pork and chicken breast fillets with near-infrared sensors [39,40,41,42]. Hence, the aim of this research was to investigate the potential of multispectral imaging, applied in a poultry processing industry, to determine the time from slaughter of four different poultry products and develop PLS-R models assessing the time from slaughter directly from spectral data. 

## 2. Materials and Methods

### 2.1. Experimental Design

Multispectral Imaging (MSI) was performed at-line in a Greek poultry industry on four different poultry products: (a) chicken breast fillets (*n* = 104, batches = 5), (b) chicken thigh fillets (*n* = 97, batches = 5), (c) chicken burger (*n* = 131, batches = 3), and (d) marinated chicken souvlaki (*n* = 144, batches = 4). At regular intervals, samples from each batch were analyzed microbiologically for the enumeration of Total Viable Counts (TVC) and *Pseudomonas* spp. in parallel with MSI spectral data acquisition. In addition, samples from each product were stored at 4 °C for 216 h (9 days), since this time period is defined by the industry as the shelf-life of the product. In parallel to the spectral acquisition, the microbiological analysis was performed simultaneously with the other batches.

Sample origins were extensive farming facilities where animals (*Gallus domesticus:* Ross strain) were fed from the company with a customized diet. Feeding consisted of grain, wheat, maize, soya bean oil and meat and premix for broilers (vitamin and mineral supplement). Chickens were slaughtered after 3 months of age and production was conducted according to the regulations of the EU 823/2004, 824/2004, 834/2004 and 543/2008. 

### 2.2. Microbiological Analysis

From each sample, 10 g was added aseptically to 90 mL of sterile quarter strength Ringer’s solution (Lab M Limited, Lancashire, United Kingdom) in a stomacher bag (Seward Medical, London, UK) and homogenized in a stomacher device (Lab Blender 400, Seward Medical, London, UK) for 60 s at room temperature. For the enumeration of Total Viable Counts (TVC) and the dominant spoilage microorganism *Pseudomonas* spp., serial decimal dilutions were prepared in the same diluent and spread on the following media: a) tryptic glucose yeast agar (Plate Count Agar, Biolife, Milan, Italy) for TVC incubated at 25 °C for 72 h, and b) Pseudomonas Agar Base with selective supplement cephalothin-fucidin-cetrimide (LabM Limited, Lancashire, UK) for *Pseudomonas* spp., incubated at 25 °C for 48 h. After incubation, colonies were enumerated and microbial counts were logarithmically transformed (log CFU/g). Poultry samples with TVC counts exceeding 7.0 log CFU/g were considered spoiled as reported elsewhere [43,44,45]. 

### 2.3. Spectra Acquisition

MSI analysis was performed using a Videometer-Lab instrument (Videometer A/S, Videometer, 2019, Herlev, Denmark) which was installed in close proximity to the production line (at-line measurement) with the possibility of sample conditioning [46]. Videometer-Lab captures surface reflectance of samples in 18 different wavelengths (405–970 nm), namely: 405, 435, 450, 470, 505, 525, 570, 590, 630, 645, 660, 700, 850, 870, 890, 910, 940 and 970 nm. Surface reflectance is recorded by a standard monochrome charged coupled device chip (CCD). The object of interest is placed at the center of an Ulbricht sphere, which has a matte white coating inside and light-emitting diodes (LEDs) with narrow-band spectral radiation positioned side by side at spheres rim. The purpose of the coating is to ensure a diffused and spatially homogenous reflectance of the sample. During instrument performance, the diodes are turned on successively leading to a monochrome image with 32-bit floating-point accuracy for each wavelength. The final outcome of MSI analysis is a data cube of spatial and spectral data for each sample of size m × n × 18 (where m × n is the image size in pixels) [47,48].

A critical point before MSI application is the assurance that the range of LEDs intensity is stable while phenomena such as shadows and object’s disfiguration are avoided [23,49]. Therefore, a light set up procedure in which the acquisition captured at zero time of the experiment (auto light) is recalled and light-emitting diodes (LEDs) intensities are stabilized. Subsequently, geometric and radiometric calibration is undertaken in the Region of Interest (ROI) area with the aim of prototype target. 

In order to exclude non-informative areas such as petri dish surface, fat, connective tissue etc., a pre-process step is required. The segmentation of ROI on the sample from no relevant areas and the implementation of Canonical Discriminant Analysis (CDA) areas is conducted via Videometer-Lab version 2.12.39 (Videometer A/S, Herlev, Denmark). Also known as Fisher (Fisher’s discriminant analysis), CDA separates pixels to different classes, based on ROI, through the following Equation (1) [50,51]:

(1) $$R\;(a)=\frac{a^{T\;}\sum_{s}a}{a^{T}\sum_{N}a}$$

where Σs is the distribution between classes and Σ_N_ is the distribution within a class.

### 2.4. Data Pre-Processing and Model Development

For the development of models estimating the time from slaughter, Partial Least Squares Regression (PLS-R) [52,53] was chosen, where spectral data were the independent variables (*n* = 36) and time from slaughter (t_s_) was the dependent variable. Time from slaughter is considered as the time elapsed from slaughter until the MSI measurement. For each poultry model, a two-stage model development approach was followed: (a) calibration and full cross-validation (using leave one out cross-validation) for model optimization and (b) external validation with samples from different batches. More specifically, for chicken breast fillets, calibration was performed using a dataset from three independent batches (*n* = 82) and external validation was undertaken with two other batches (*n* = 22). Similarly, the PLS-R model for chicken thigh fillets was constructed using a training dataset from three batches (*n* = 67), whereas two other batches (*n* = 30) were used to assess the prediction performance of the model. Concerning the chicken burger, two batches (*n* = 87) were used in model training and one batch (*n* = 44) in prediction. Finally, for marinated chicken souvlaki, the dataset consisted of two batches (*n* = 91) for training and two different batches (*n* = 43) for external validation. 

Prior to analysis, spectral data for each type of poultry product were pre-processed by different transformation techniques in an attempt to reduce random or systematic variations [22,54]. Reducing the total volume of data results in effective multispectral imaging systems and image acquisition with relatively low spatial resolutions in a few important wavelengths [13]. Standard Normal Variate transformation (SNV) Equation (2) was applied in the case of chicken thigh and burger in order to avoid collinear and “noisy” data areas [55]. In contrast, spectral data from chicken breast and marinated souvlaki were pre-processed with baseline offset treatment [56,57] Equation (3). Regarding time from slaughter (y variable), a logarithmic transformation was considered necessary due to large differences in the intensities of the raw data [58]. Data pre-treatment, model development and validation were implemented using the Unscrambler© v.9.7 software (CAMO Software AS, Oslo, Norway).

(2) $$s_{I}^{\mathrm{SNV}}=\frac{S_{I}-\mathrm{mean}\left(S\right)}{\mathrm{stdev}\left(S\right)}$$

where S refers to pixel-wise spectra, S_i_ is the “old” information contained in a specific wavelength and Si^SNV^ is the “new-transformed” information contained in a specific wavelength [22].

S(x) = x − min (x)(3)

where S denotes pixel-wise spectra for one sample, x is information contained at a specific wavelength, and min(x) is the minimum variable in the x dataset.

## 3. Results

### 3.1. Microbiological Analysis

**Figure 1:**
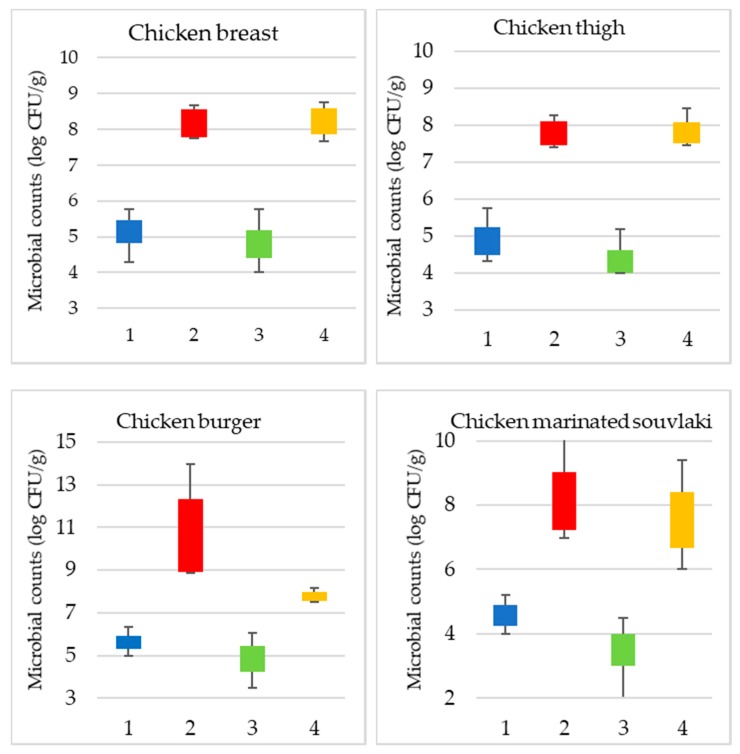
Boxplots for microbial counts (log CFU/g) of TVC (1: blue, 2: red) and *Pseudomonas* spp. (3: green, 4: orange) in fresh (1: blue, 3: green) and spoiled (2: red, 4: orange) samples of each product.

Τhe range of the microbial population of TVC and *Pseudomonas* spp. for the different batches of poultry products and storage time is illustrated in Figure 1. Additionally, the spread of TVC counts for fresh and spoiled samples is provided for each product case. More specifically, for chicken breast fillets, the initial number of TVC and *Pseudomonas* spp. was 5.2 (±0.6) and 4.9 (±0.82) log CFU/g respectively, whereas in spoiled samples the respective values were 8.4 (±0.46) and 8.3 (±0.47) log CFU/g. Additionally, for chicken thigh fillets, samples were considered fresh with TVC and *Pseudomonas* spp. counts at 5 (±0.83) and 4.5 (±0.98) log CFU/g. Spoiled chicken thigh samples had TVC and *Pseudomonas* spp. values at 7.9 (±0.50) and 7.8 (±0.49) log CFU/g, respectively. 

For chicken burger samples, TVC and *Pseudomonas* spp. counts in fresh samples were 5.5 (±0.36) and 4.8 (±0.75) log CFU/g, respectively, while in spoiled samples the respective counts were 10.7 (±1.9) and 7.8 (±0.25) log CFU/g. Finally, marinated chicken souvlaki fresh samples had TVC and *Pseudomonas* spp. counts at 4.6 (±0.50) and 3.6 (±0.71) log CFU/g, respectively. In contrast, TVC and *Pseudomonas* spp. values for spoiled samples were 7.9 (±0.95) and 7.5 (±1.00) log CFU/g, respectively.

Results from storage experiments at 4 °C showed that chicken breast fillets were determined as spoiled beyond 168 h of storage (TVC > 7 log CFU/g) with TVC value at 7.76 log CFU/g and *Pseudomonas* spp. counts at 7.6 log CFU/g. For chicken thigh fillets, samples were characterized as fresh until 120 h when TVC and *Pseudomonas* spp. counts were 7.5 log CFU/g and 7.5 log CFU/g, respectively. TVC and *Pseudomonas* spp. counts were 8.8 log CFU/g and 7.7 log CFU/g in spoiled chicken burger samples (storage time 216 h). Moreover, chicken marinated souvlaki was defined as spoiled after 168 h of storage in which TVC were 7.3 log CFU/g and *Pseudomonas* spp. counts were 6.8 log CFU/g.

### 3.2. Spectral Measurements

**Figure 2:**
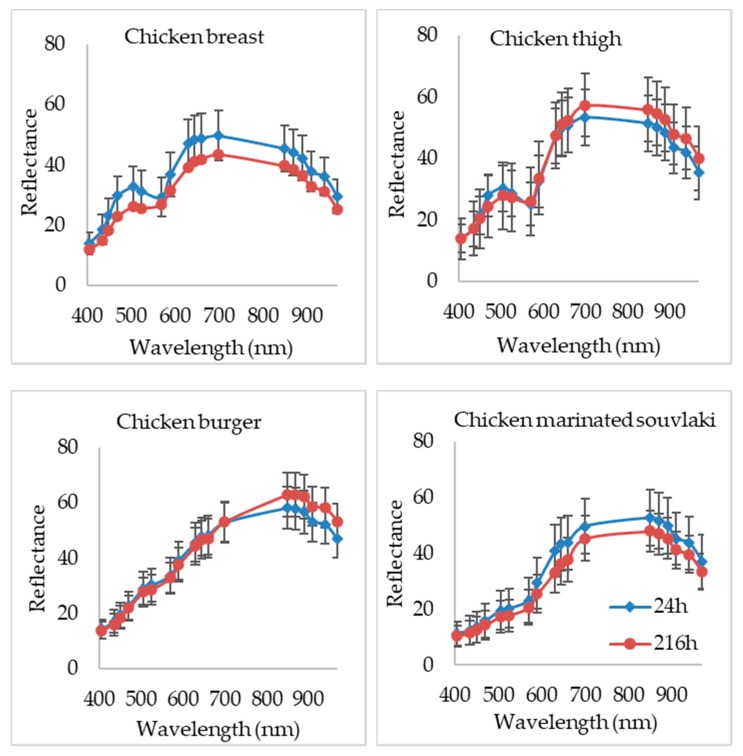
Spectra from MSI analysis (405–970 nm) for each poultry product at 24 h (blue line) and 216 h (red line) of storage at 4 °C.

For the development of PLS-R models, each wavelength contributed differently to each category of poultry product, despite the fact that all these products have the same basic ingredient (i.e., poultry meat). This is demonstrated in Figure 2 where differences could be observed in the spectra between each type of product during storage at 4 °C, based mostly on their nutrition composition difference [59,60].

The same figure (Figure 2) confirms also the ability of this spectroscopic method to detect and/or separate spoiled from fresh samples for each of the four products. For instance, in the case of chicken breast, wavelengths with variations in reflectance for fresh and spoiled samples were located in the areas of 470–570 nm and 590–970 nm, respectively. Wavelength range from 660 to 970 nm seemed to affect the estimation of spoilage for chicken thigh. Similarly, for marinated chicken souvlaki wavelengths above 570 nm deviated between fresh and spoiled samples, whereas for the chicken burger 850–970 nm were noticed as different.

### 3.3. PLS-R Model Performance

**Figure 3:**
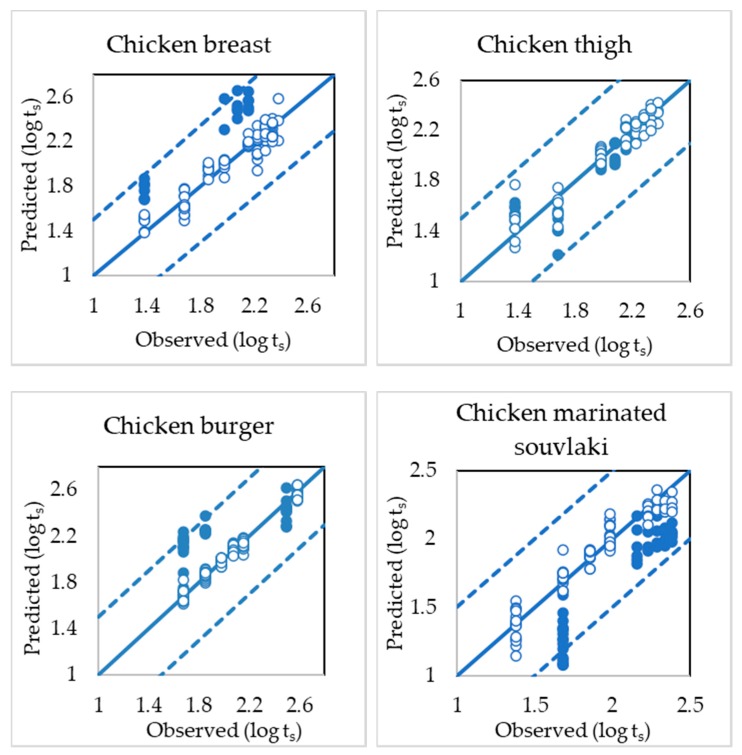
Comparison of observed (open symbols) and predicted (solid symbols) values of time from slaughter (log t_s_) after the development of the PLS-R model. Solid line depicts the line of equity (y = x) and dashed lines are ± 1.6 log t_s_ (i.e., 48 h after slaughter).

RLS-R models assessing the time from slaughter showed satisfactory performance for each category of poultry product as inferred both graphically (Figure 3) and computationally based on performance indices such as slope, offset, correlation coefficient (r) and root mean squared error (RMSE) (Table 1).

For chicken breast fillets, time from slaughter was estimated quite accurately despite the variations between batches, with r_p_ and RMSE_p_ values for the prediction dataset of 0.886 and 0.383, respectively. Differences between batches and ingredients (spices, herbs and sauce) used in marinated chicken souvlaki did not affect the prediction performance of the PLS-R model, with r_p_ and RMSE_p_ values of 0.934 and 0.348, respectively. Even though chicken thigh muscle has a more complex texture, with a higher percentage of fat and connective fat tissue [61,62], no differences were observed between batches and subsequently, external validation was performed satisfactorily with r_p_ and RMSE_p_ values of 0.859 and 0.160, respectively. Similarly, the presence of vegetables (peppers, onions and herbs) and spices in the homogeneous mixture of chicken burgers was not an obstacle for the external validation, where r_p_ and RMSE_p_ values were 0.778 and 0.285, respectively. The above-mentioned RMSE values of prediction indicate satisfactory accuracy of the models used to assess the observed data [27,63].

**Figure 4:**
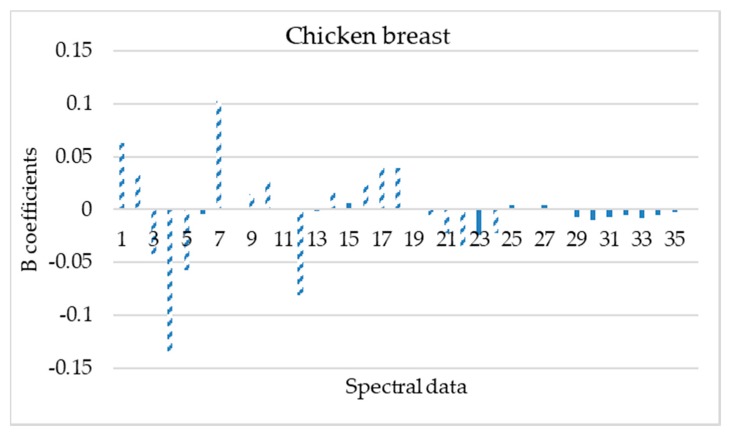
Spectral data (mean and standard deviation) influence (b coefficients) on PLS-R model construction for chicken breast samples. Dashed bars represent data that influenced more the model (1,19: 405 nm; 2, 20: 435 nm; 3, 21: 450 nm; 4, 22: 470 nm; 5, 23: 505 nm; 6, 24: 525 nm; 7, 25: 570 nm; 8, 26: 590 nm; 9, 27: 630 nm; 10, 28: 645 nm; 11, 29: 660 nm; 12, 30: 700 nm; 13, 31: 850 nm; 14, 32: 870 nm; 15, 33: 890 nm; 16, 34: 910 nm; 17, 35: 940 nm and 18, 36: 970 nm).

**Figure 5:**
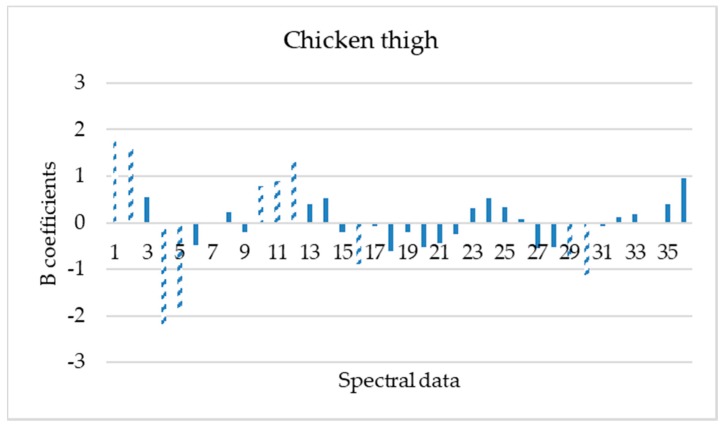
Spectral data (mean and standard deviation) influence (b coefficients) to PLS-R model construction for chicken thigh samples. Dashed bars represent data that influenced more the model (1, 19: 405 nm; 2, 20: 435 nm; 3, 21: 450 nm; 4, 22: 470 nm; 5, 23: 505 nm; 6, 24: 525 nm; 7, 25: 570 nm; 8, 26: 590 nm; 9, 27: 630 nm; 10, 28: 645 nm; 11, 29: 660 nm; 12, 30: 700 nm; 13, 31: 850 nm; 14, 32: 870 nm; 15, 33: 890 nm; 16, 34: 910 nm; 17, 35: 940 nm and 18, 36: 970 nm).

**Figure 6:**
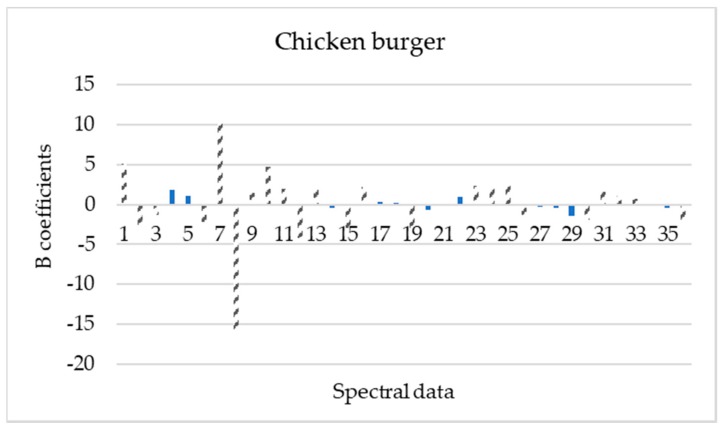
Spectral data (mean and standard deviation) influence (b coefficients) to PLS-R model construction for chicken burger samples. Dashed bars represent data that influenced more the model (1, 19: 405 nm; 2, 20: 435 nm; 3, 21: 450 nm; 4, 22: 470 nm; 5, 23: 505 nm; 6, 24: 525 nm; 7, 25: 570 nm; 8, 26: 590 nm; 9, 27: 630 nm; 10, 28: 645 nm; 11, 29: 660 nm; 12, 30: 700 nm; 13, 31: 850 nm; 14, 32: 870 nm; 15, 33: 890 nm; 16, 34: 910 nm; 17, 35: 940 nm and 18, 36: 970 nm).

**Figure 7:**
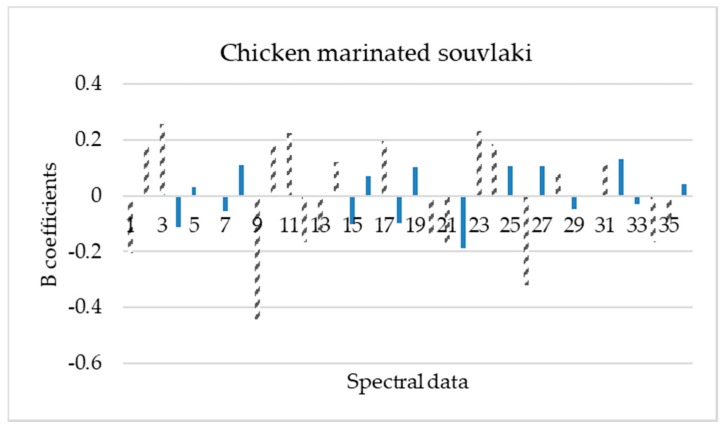
Spectral data (mean and standard deviation) influence (b coefficients) to PLS-R model construction for chicken marinated souvlaki samples. Dashed bars represent data that influenced more the model (1, 19: 405 nm; 2, 20: 435 nm; 3, 21: 450 nm; 4, 22: 470 nm; 5, 23: 505 nm; 6, 24: 525 nm; 7, 25: 570 nm; 8, 26: 590 nm; 9, 27: 630 nm; 10, 28: 645 nm; 11, 29: 660 nm; 12, 30: 700 nm; 13, 31: 850 nm; 14, 32: 870 nm; 15, 33: 890 nm; 16, 34: 910 nm; 17, 35: 940 nm and 18, 36: 970 nm).

The important wavelengths (mean values and standard deviations) reflecting the characteristics of spectral data for each poultry product were obtained based on the beta regression coefficients (Figure 4, Figure 5, Figure 6 and Figure 7).

Based on these beta regression coefficients, equations were constructed for the assessment of time from slaughter for each product (Equations (4)–(7)).

**Y_ts,chicken breast_** = 2.016 + 0.063 X_mean,405 nm_ + 0.033 X_mean,435 nm_ − 0.042 X_mean,450 nm_ − 0.134 X_mean,470 nm_ − 0.057 X_mean,505 nm_ + 0.103 X_mean,570 nm_ + 0.015 X_mean,630 nm_ + 0.027 X_mean,645 nm_ − 0.081 X_mean,700 nm_ + 0.012 X_mean,870 nm_ + 0.023 X_mean,910 nm_ + 0.040 X_mean,940 nm_ + 0.039 X_mean, 970 nm_ − 0.022 X_SD,450 nm_ − 3.505 X_SD,470 nm_ − 2.462 X_SD,505 nm_ − 0.023 X_SD,525 nm_ (4)

**Y_ts,chicken thigh_** = −1.287 + 1.823 X_mean,405 nm_ + 1.596 X_mean, 435 nm_ − 2.277 X_mean, 470 nm_ − 1.835 X_mean,505 nm_ + 0.774 X_mean,645 nm_ + 0.901 X_mean, 660 nm_ + 1.407 X_mean, 700 nm_ − 0.888 X_mean, 910 nm_ − 0.754 X_SD, 660 nm_ − 1.135 X_SD, 700 nm_ (5)

**Y_ts,chicken burger_** = 3.042 + 5.092 X_mean,405 nm_ − 2.948 X_mean,435 nm_ − 1.332 X_mean,450 nm_ − 2.205 X_mean,525 nm_ + 10.153 X_mean,570 nm_ − 15.754 X_mean,590 nm_+ 1.397 X_mean,630 nm_ + 4.716 X_mean,645 nm_ + 1.982 X_mean,660 nm_ − 4.230 X_mean,700 nm_ + 2.344 X_mean,850 nm_ − 2.989 X_mean,890 nm_ + 2.237 X_mean,910 nm_- 3.283 X_SD,405 nm_ +2.382 X_SD,505 nm_ + 2.161 X_SD,525 nm_ + 2.304 X_SD,570 nm_ − 1.799 X_SD,590 nm_ −1.402 X_SD,660 nm_ − 1.874 X_SD,700 nm_ +1.558 X_SD,850 nm_ + 1.112 X_SD,870 nm_ −2.188 X_SD,970 nm_ (6)

**Y_ts,chicken marinated souvlaki_** = 3.071 − 0.205 X_mean,405 nm_ + 0.180 X_mean,435 nm_ + 0.255 X_mean,450 nm_ − 0.442 X_mean,630 nm_ + 0.189 X_mean,645 nm_ + 0.223 X_mean,660 nm_ − 0.168 X_mean,700 nm_ − 0.122 X_mean,850 nm_ + 0.119 X_mean,870 nm_ + 0.196 X_mean,940 nm_ − 0.150 X_SD,435 nm_ − 0.185 X_SD,450 nm_ + 0.232 X_SD,505 nm_ + 0.184 X_SD,525 nm_ − 0.319 X_SD,590 nm_ + 0.091 X_SD,645 nm_ + 0.131 X_SD,870 nm_ − 0.165 X_SD,910 nm_ − 9.849 X_SD,940 nm_ (7)

## 4. Discussion

Microbiological analysis demonstrated variations between batches for each category of poultry product even though the examined samples were obtained by the same farming conditions, slaughter process and production. For chicken breast and thigh samples, differences occurred at the initial microbial load (TVC and *Pseudomonas* spp.) based mostly on animal strain and fat content [62]. Furthermore, in the case of processed poultry products, the presence of additional ingredients such as vegetables and herbs seemed to influence the initial and final load of microorganisms.

MSI acquisition showed variations in reflectance at many wavelengths between the four poultry products due to their differences in the food matrix (chicken breast and chicken thigh) and the supplementary ingredients used in the production process of different chicken products (i.e., burger and marinated souvlaki). Moreover, spectra figures for fresh and spoiled samples (Figure 2) provided by MSI application indicated reflectance differences at several wavelengths, which are firmly linked to biochemical alterations and metabolic compounds produced by the spoilage microbiota on the surface of meat and poultry products. More specifically, reflectance at 570–700 nm is related to respiratory pigments such as myoglobin (570 nm), oxymyoglobin (590 nm) and metmyoglobin (630 nm) [23,24,59]. In the NIR region, absorption bands at 910 nm are linked to denaturation of proteins [60,64] while at 750 and 970 nm, O-H second overtones are related to the moisture content in the samples [26,38,53]. In addition, absorption bands observed in the NIR region (928 and 940 nm) are correlated to the presence of fatty acids and fat within the sample matrix [14,60,65].

PLS-R models predicted satisfactorily the time from slaughter for each poultry product (Table 1) where the chicken thigh model showed the lowest value of RMSE followed by the chicken burger model. RMSE and r values of prediction were in the range of 0.160–0.348 and 0.778–0.943 respectively, for all PLS-R models. Model performance was gradually deteriorated from the calibration to the prediction stage. As illustrated in Figure 3, batches used in external validation differed from the calibration dataset in all products and especially in the case of the chicken breast. These findings indicate the importance of performing validation with independent datasets (batches at different time points) and to include as much variability as possible in the developed model [64,66,67]. Additionally, the developed model addressed for at-line implementation must be validated by an independent dataset in order to construct an accurate and robust model [24,52]. Despite this variation in batches, both calibration and prediction datasets in Figure 3 are situated within the limit area of ±1.6 log t_s_ resulting in acceptable PLS-R models. For chicken marinated souvlaki and burger models, variations between batches and higher RMSE values could be explained due to different types of ingredients such as spices, chopped vegetables and marinade employed in the production process.

Beta regression coefficients revealed the influence of each wavelength on the assessment of time from slaughter for each poultry product. According to Figure 4, Figure 5, Figure 6 and Figure 7, wavelengths with high positive or negative values have an important contribution to the model and convey useful information. The comparison of these findings with the raw spectra shown in Figure 2 confirms the significant role of reflectance bands in the range 570–700 nm and 700–970 nm for the development of PLS-R models [38,68]. As mentioned above, absorption bands at NIR region of 910 nm seemed to be associated with proteins, which are in abundance in chicken meat, especially in chicken breast [61]. The influence of muscle pigments and water content on the classification of chicken breast fillets was also highlighted by Yang et al., where samples were successfully classified in different quality grades [25].

In conclusion, the performance of the developed PLS-R models showed an efficient prediction of the time from slaughter (t_s_) for each poultry product. The food matrix (muscle type, spices and marinade) had a great impact on the prediction of time from slaughter with marinated chicken souvlaki and chicken thigh models showing satisfactory performance. The prediction was also influenced by the variations between batches. Furthermore, the selection of different datasets (batches) as external validation assures the efficient prediction of models. Additional measurements and continuous information feedback could ameliorate the existing models and result in a more successful prediction of time from slaughter.

## Figures and Tables

**Table 1:** Performance indices (slope, offset, r and RMSE) for PLS-R model development and validation for each poultry product.

Poultry Product	Stage of Modelling	No of Samples	Slope	Offset	r (Correlation Coefficient)	RMSE
Chicken Breast	Calibration	82	0.933	0.138	0.966	0.076
	FCV ^1^	82	0.916	0.173	0.953	0.091
	Prediction	22	1.150	0.055	0.886	0.383
Chicken Thigh	Calibration	67	0.953	0.097	0.976	0.065
	FCV	67	0.933	0.136	0.957	0.088
	Prediction	30	0.854	0.243	0.859	0.160
Chicken Burger	Calibration	87	0.982	0.035	0.991	0.033
	FCV	87	0.968	0.063	0.987	0.040
	Prediction	44	0.513	1.172	0.778	0.285
Chicken Marinated Souvlaki	Calibration	91	0.962	0.073	0.981	0.067
	FCV	91	0.954	0.092	0.964	0.093
	Prediction	43	1.183	0.650	0.934	0.348

^1^ FCV: Full cross-validation.
